# The Local Honorary Treasurer

**Published:** 1918-10-05

**Authors:** 


### THE MATRONS' AND SISTERS' DEPARTMENT.

### THE LOCAL HONORARY TREASURER.

The financial side of the Local Centres has been enormously facilitated by the decision of the College of Nursing to charge no annual subscription. Very few nurses, it may be surmised, would prove equal to the strain of paying double fee every year to the Local Centre and to Headquarters. The fatal misgiving which finds utterance in the familiar cry, "What is the good of it?" would inevitably haunt their minds after the first enthusiastic payments had been made. But as things are, the Local Honorary Treasurer has the first and only claim on the members' business instincts, and to judge from the amount of the subscription imposed by most of the Centres she will not be called on to plunge very deep into their pockets. All the same, there are special difficulties in the way of collecting small subscriptions, and the measure of success which attends their retrieval spells the measure of success enjoyed by the Centre.

There is no doubt that the Local Honorary Treasurer ought to have a tinge of business education. If she lack it entirely she should take a few lessons, for even in dealing with half-crowns (we might say especially in dealing with these little sums), business knowledge is indispensable. She must recognise from the outset that subscriptions will not be paid unless they are asked for. It is not the slightest use appealing to the loyalty, or good feeling, or any other quality the nurse may possess—or lack—to ensure punctual payment every year. The nurse will agree in theory, but in practice, as in the case of the Junius S. Morgan Benevolent Fund, many a pay-day will find her oblivious of its recurrence. She will *mean* to pay as soon as she gets a reminder, but not improbably it may take two postcards to bring her to the point. It is most important that the reminder sent should be courteous and cordial in tone. It may be read by others besides the recipient, and there must be no insinuation that the member is forgetful, mean, or irritating. It would save much trouble and postage if a correspondent could be found in all the institutions and hostels most frequented by members, who would collect subscriptions for the Treasurer every year. When this is done, however, the Treasurer should herself send the official receipt, so as to keep in touch with the members. A supplementary book of interim receipts and counterfoils should be issued to every correspondent who assists in this way, and the counterfoils should be sent up every year for the inspection of the auditor.

The ordinary receipts of the Local Centre will fall under the head of Members' Subscriptions; Annual Subscriptions (non-members); Donations; Receipts from Lectures, etc. We do not include in these subscriptions and donations any collected through the agency of the Finance Committee, which are in an entirely different category and will be considered later on. But both outsiders and members will certainly testify their interests by gifts and they will be needed if the Centre is to be launched on a satisfactory basis. It is doubtful whether the half-crown subscription, at any rate during the present conditions as regards printing and postage, can suffice to meet the preliminary expenses. A few office requisites will be needed by Treasurer and Secretary, and some outlay there must be in bringing members together.

It is very necessary that the Treasurer should analyse all expenditure and keep the items distinct in her petty-cash book. Postage, printing, stationery, hire of rooms, lecturers' fees, and other items will vary in their demands on the exchequer according to the circumstances of the Centre. In places where nearly all the members reside within easy distance, where rooms free of charge are available for meetings, where lecturers offer their services gratuitously, where voluntary help is forthcoming to duplicate circulars and so save printing, the expenses will be minimised. But whatever the outlay may be, whether it be more than was anticipated and the receipts unexpectedly small, or whether there be a good balance, the exact condition of affairs ought to be imparted by the Executive Committee every year to the whole body of members at a general meeting. We know of one literary society in which the treasurer never issues a statement of accounts because she declares she would get no more annual subscriptions if it were known she had a few pounds balance in hand. This kind of secretiveness is not only silly, it verges on dishonesty. Every subscribing member is entitled to receive an annual statement of accounts however small the turnover may be.

It is a matter for time to decide whether the Local

Centres can so effectually finance themselves as to be capable of sending a percentage of their subscriptions as a contribution to the parent College of Nursing on which they are dependent for their existence. As a matter of principle it is highly desirable that this course should be adopted. Even if the amount were no more than 20 per cent. of the subscription, or sixpence per head, every Centre would benefit by the welding of a strong link with Headquarters, and the College would be at no loss to find a special object, of interest to all parts of the kingdom, to which sums, yielded by the local levy could be devoted, and which the Local Centres would in time support entirely.

